# Turning decisions into actions

**DOI:** 10.1371/journal.pbio.3001927

**Published:** 2022-12-16

**Authors:** Alexander Gail

**Affiliations:** 1 German Primate Center – Leibniz Institute for Primate Research, Göttingen, Germany; 2 University of Göttingen, Faculty of Biology and Psychology, Göttingen, Germany

## Abstract

Are selection and control of action serial processes of separate neural modules? This Primer explores a study in PLOS Biology which argues against this and in favor of an integrated process distributed over multiple brain regions, each contributing in a distinct way.

## Teaser

Are selection and control of action serial processes of separate neural modules? A new study in *PLOS Biology* argues against this and in favor of an integrated process distributed across multiple brain regions, each contributing in a distinct way.

We take action once a decision has been reached. Such serial modular processing is what most people probably experience by introspection and how classical models of decision making are set up. So why is it that brain regions that are associated with the planning and control of goal-directed movements are modulated by various decision-related variables, like the sensory evidence for or against a choice or its expected reward [1,2], and reflect biased competition between action alternatives [3,4,5]? In a new study, Thura and colleagues [6] address this question of functional modularization versus overlap in decision-making and sensorimotor control by systematically comparing three regions in the cerebral cortex and two nuclei of the basal ganglia in rhesus monkeys during the same decision-making task. They conclude that the studied regions are part of a large-scale integrated dynamic system that governs both action selection and control, while at the same time making distinct functional contributions to the overall task of reaching a decision.

For most of primate evolution, decisions were not about selecting the most affordable mobile phone contract or the best university to study at, cases in which collection and weighing of evidence for the different options (deliberation) and selection of the preferred option tend to occur separately from the according actions. Instead, most daily decisions closely tie to immediately pending or already ongoing actions. Such sensorimotor decisions are vital for most animal species, for example, when chasing pray, escaping predators, or foraging in a group.

Given the ecological relevance, it should not be surprising that many regions of the primate brain contribute to choice behavior. Yet, the very nature of each brain region’s contribution is still unclear. One challenge arises from the many different decision-relevant variables that modulate neural responses in these association regions of the brain, leading to mixed selectivity of individual neurons. Qualifying these neurons’ roles via functional dependency from a single, most important stimulus or movement parameter (“tuning”), as was highly successfully done for decades in the peripheral sensory or motor structures of the brain, explains only small fractions of the observed neural responses. Another challenge results from a tradition of treating decision-making as a cognitive selection process, mostly among discrete choices, while conceptualizing movement execution as a feedback-dependent continuous adaptive control mechanism. These two concepts led to vastly different and independent models for the two aspects of behavior, whereas more recent studies have suggested that optimization principles might partially overlap between decision-making and movement control [7,8].

In their current study, Thura and colleagues [6] use a dynamic systems approach to partly overcome the above challenges, thereby making an exciting and important step towards a more unified approach to the neurobiology of deciding and acting. With an elegant task design, they experimentally controlled the difficulty and the urge of committing to one of two reach targets by successively adding (or subtracting) evidence for and against either of two options, varying the timing parameters of the task to encourage adjustment of the speed-accuracy trade-off between blocks of trials. This design helps to distinguish between decision-relevant cognitive variables, like urgency, deliberation, or commitment to a choice. In a series of experiments, the authors collected data from many individual neurons across brain regions while rhesus monkeys performed the task and then analyzed the data with a dynamic systems approach in which the response of each neuron spanned one dimension of a high-dimensional space and the combined time-varying responses of all neurons described a trajectory in this space. Dimensionality reduction allowed recombining and sorting of the dimensions of this space to extract the components of the neural activity that explain most of its dynamics. The results were surprisingly intuitive.

Thura and colleagues [6] elaborate beautifully how individual components of neural activity relate to specific decision variables. One prominent component rises steeply when animals transition between deliberation and commitment to a choice, irrespective of how difficult or urgent the decision is. Other prominent components prior to commitment indicate the strength of momentary evidence for either response option (at least when there is enough such evidence), the increasing urgency to commit to a response over time, or the slow and fast decision contexts giving rise to lower or higher urgency.

**Fig 1:**
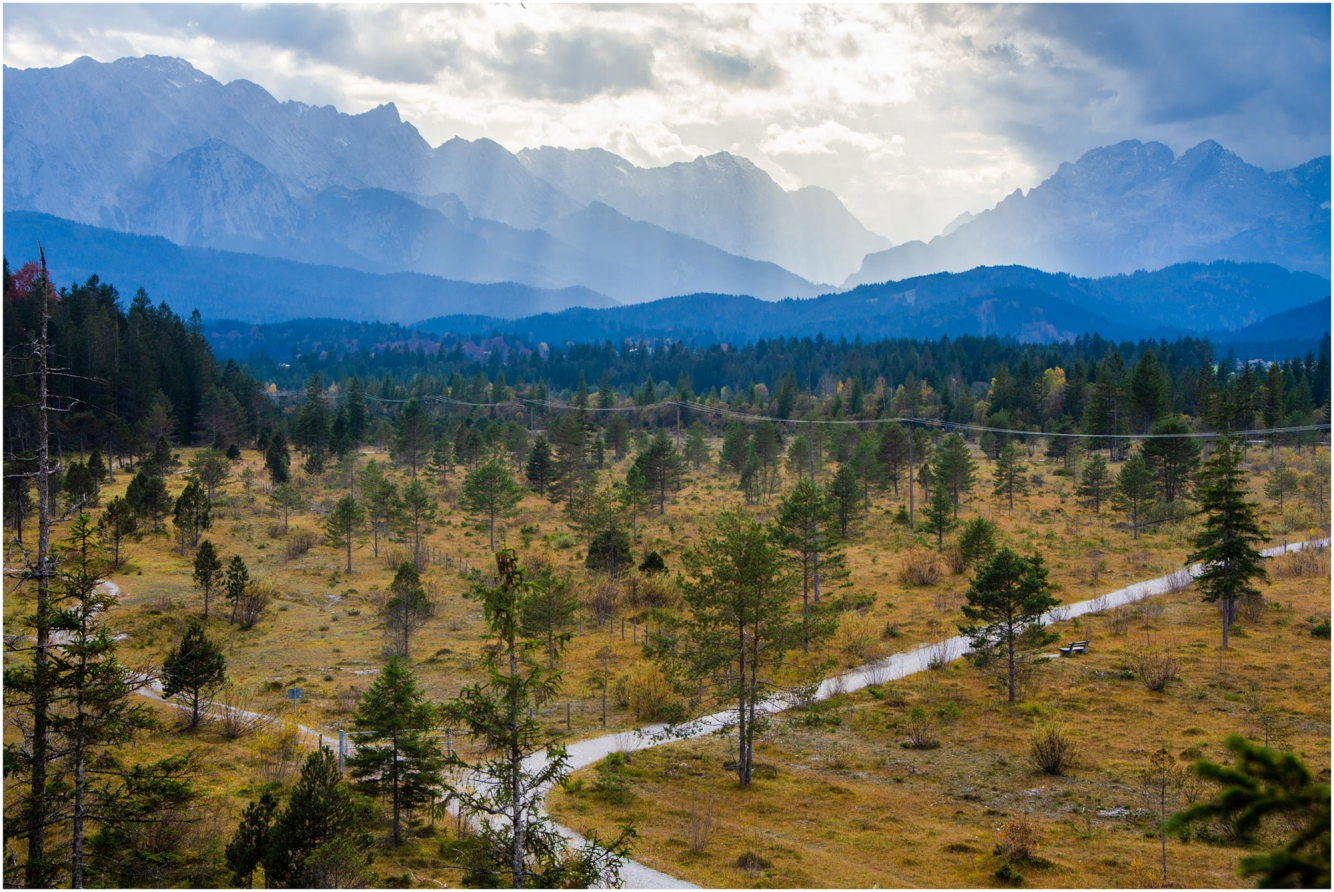
Deciding while acting. Should I take the well-paved road towards the darker clouds or the more strenuous rougher track with lower risk of rain? With enough time for deliberation, one could research track conditions and the weather forecast to achieve optimal choice. But when going downhill at a fast pace, the intersection comes closer quickly and the urge to commit to one of the two paths before having collected all possible evidence grows. Similarly, deciding and acting in a tightly integrated fashion is an important survival skill for primates and most other animals during ecologically relevant behaviors. The unifying framework provided by Thura and colleagues [6] describes the underlying neurophysiological processes as a distributed dynamic system composed of different cortical and subcortical regions of the primate brain, where different regions contribute specific aspects of choice behavior, like the evidence in favor of either option or the urgency to decide.

The brain regions under study thereby show overlapping patterns but also clear differences during deliberation. The dorsolateral prefrontal cortex (dlPFC), for example, mostly reflects evidence in favor of either of two response options, while the globus pallidus (GP) externus and internus primarily indicate urgency-related parameters. The authors suggest that dlPFC and GP signals get integrated in the dorsal premotor cortex and primary motor cortex, where a biased competition between candidate actions is implemented. During deliberation, this competition is comparably weak, allowing for the coexistence of multiple candidate actions, whereas an increasingly stronger “winner-takes-all”-like competition, driven by the subcortical urgency input, leads to commitment and sets the stage for evolution of the neural dynamics into a specific movement associated with the selected action. The situation of the neural dynamics is similar to the landscape in Fig 1: While on an open plane, one can easily change direction as evidence emerges (observed during deliberation); once entering a specific valley in the strongly modulated mountain range, the path narrows and its continuation is set (observed after commitment and during control of movement). Importantly, deliberation and control of movement mark different dynamic modes but are supported by the same recurrent network of neurons.

The finding of overlapping neural substrates for action selection and control in sensorimotor structures of the frontal lobe, complemented by urgency signals from subcortical structures, are nicely consistent with the urgency-gating model of decision-making that was previously proposed by this group [9]. While individual findings might not be new, the dynamic systems approach taken here systematically acknowledges the inherently dynamic nature of neural networks and provides a convincing integrated account for the processes of selecting and controlling actions as part of a single recurrent system. The comparative view of a significant number of frontal lobe and subcortical regions highlights their idiosyncratic contributions to most relevant cognitive aspects of decision-making. The role of very similar neural findings observed in parietal sensorimotor structures [5,10] remains less clear from the current discussion. Future expansion of the concept to illuminate mutual roles of parietal and premotor regions will be interesting. Also, while the overlap in neural substrates is undisputed, it can be debated if the observation of two different dynamic modes before and after commitment to a choice categorically rejects the idea of two distinct serial modules for selection and control of action. This might be difficult to test with behavioral tasks that impose a serial structure of first deliberating and then acting on the animal’s behavior. In this sense, one might be able to make an even stronger case for the proposed framework when studying deciding while acting, i.e., by demonstrating that the neural results generalize to situations characterized by simultaneous selection and control of action, as common for natural behaviors. Clearly, the presented framework provides an excellent basis to integrate such future findings.
